# Sparse Angle CBCT Reconstruction Based on Guided Image Filtering

**DOI:** 10.3389/fonc.2022.832037

**Published:** 2022-04-27

**Authors:** Siyuan Xu, Bo Yang, Congcong Xu, Jiawei Tian, Yan Liu, Lirong Yin, Shan Liu, Wenfeng Zheng, Chao Liu

**Affiliations:** ^1^School of Automation, University of Electronic Science and Technology of China, Chengdu, China; ^2^Department of Geography and Anthropology, Louisiana State University, Baton Rouge, LA, United States; ^3^Laboratoire d'Informatique, de Robotique et de Microélectronique de Montpellier (LIRMM), Unité Mixte de Recherche (UMR) 5506, French National Center for Scientific Research (CNRS) - University of Montpellier (UM), Montpellier, France

**Keywords:** CBCT reconstruction, guided image filtering, simultaneous algebraic reconstruction technique, the total p-Variation minimization, radiation therapy

## Abstract

Cone-beam Computerized Tomography (CBCT) has the advantages of high ray utilization and detection efficiency, short scan time, high spatial and isotropic resolution. However, the X-rays emitted by CBCT examination are harmful to the human body, so reducing the radiation dose without damaging the reconstruction quality is the key to the reconstruction of CBCT. In this paper, we propose a sparse angle CBCT reconstruction algorithm based on Guided Image FilteringGIF, which combines the classic Simultaneous Algebra Reconstruction Technique(SART) and the Total p-Variation (TpV) minimization. Due to the good edge-preserving ability of SART and noise suppression ability of TpV minimization, the proposed method can suppress noise and artifacts while preserving edge and texture information in reconstructed images. Experimental results based on simulated and real-measured CBCT datasets show the advantages of the proposed method.

## 1 Introduction

Computerized Tomography (CT) technology has attracted widespread attention in the field of medical imaging technology since 1963. Unlike conventional CT, the Cone-beam Computerized Tomography (CBCT) system uses a flat panel detector, so it can reconstruct a three-dimensional CT image after only one scan with high ray utilization and short scanning time. Moreover, CBCT images have higher spatial resolution, and the imaging effect has isotropic resolution in three-dimensional space. Because of these advantages, CBCT has become more and more important in radiation therapy. Despite the above-mentioned advantages of CBCT, X-rays are still harmful to the body. Therefore, the study of incomplete angle CBCT reconstruction algorithm is of great significance for reducing radiation dose, improving the quality of reconstruction, and the development and application of CBCT.

The traditional reconstruction algorithm of CBCT is mainly divided into analytical methods and iterative methods. The FDK algorithm (1) is a classic analytical method proposed by Feldkamp, which based on the filtered back-projection algorithm. FDK is suitable for the reconstruction of circular trajectory CBCT. Since no iterative calculation is required, the calculation required by FDK is low and easy to implement. In 2005, Tang et al. proposed a 3D weighted cone beam filter back projection (CB-FBP) (2) algorithm based on the FDK. In CB-FBP, the projection data is weighted before the 3D back-projection to reduce the inconsistency of the conjugate rays. In 2006, the CB-FBP is extended in spiral scan CT (3).

In 2006, Donoho proposed a theory of Compressed Sensing (CS) (4), which provides a new way for solving the reconstruction problem of sparse angle CBCT. For a sparse sampled signal, even if the sampling frequency is much smaller than the Nyquist sampling frequency, the sparse solution of the underdetermined linear equation can be solved and the original signal can be restored with the CS techniques. The sparse optimization theory based on CS mainly involves two aspects (5), sparse representation model and optimization algorithm.

So far, the Total Variation (TV) minimization model is the most widely used in the reconstruction of sparse angle CBCT. In (6), Candes et al. proposed a constrained TV minimization model based on the theory of CS, assumed that the Gradient Magnitude Image (GMI) is sparse, added the l_1_-norm minimization of GMI into the regularization constraints, and achieved a more accurate reconstruction result in image reconstruction. In the same year, Sidky et al. (7) applied TV minimization to limited-angle divergent-beam CT, and achieved good reconstruction results. In 2008, they further improved the TV minimization model, and proposed the Adaptive Steepest Descent-Projection onto Convex Sets (ASD-POCS) algorithm (8). which uses convex set projection to enforce constraints, and minimizes TV through the steepest descent method with adaptive step size. The ASD-POCS is relatively simple, and can effectively suppress cone beam artifacts, but it has a long solution time, and it is difficult to balance convex set projection and steepest descent. To overcome these shortcomings, some improved algorithms have been proposed. Liu et al. (9) proposed an adaptive weighted TV minimization algorithm, which takes the anisotropic edge characteristics between adjacent image voxels into consideration, and adaptively adjusts the preserved edge details with local image gradients. In (10), Bian et al. proposed an adaptive steepest descent weighted convex set projection algorithm. Zhang et al. (8) proposed an alternating direction TV minimization algorithm, which reformulates the TV problem as a linear equality constrained separable problem of the objective function, and splits the augmented Lagrangian function minimization into two sub-problems. In 2014, Cai et al. (11) proposed an edge-guided reconstruction algorithm based on weighted alternating direction minimization, which combined TV regularization and iterative edge detection strategies. Wang et al. (12) developed a distributed reconstruction algorithm based on Alternating Direction Minimization(ADM) for TV minimization, which accelerated the reconstruction speed without losing accuracy. In 2015, they further proposed a general block distribution reconstruction algorithm based on TV minimization and ADM algorithm (13), solved the need for large-scale reconstruction with few angles.

Due to the segmental constant assumption of TV minimization, the TV regularization method penalizes the image gradient, and the reconstruction results were locally too smooth, leading to a step effect (14). In order to overcome the step effect, many improved TV algorithms were proposed. Tian et al. (15) proposed edge-preserving TV, by introducing penalty weights into the original TV regularization model. Chen et al. (16) proposed an anisotropic TV minimization algorithm to balance the smoothness and data consistency caused by TV minimization. Liu et al. (17) proposed a TVS-POCS algorithm for projecting TV onto a convex set.

In 2010, Yang et al. (18) assumed the target region (Region of Interest, ROI) was a piecewise constant, allowed piecewise polynomials, and introduced high-order TV (HTV) minimization to solve internal problems. Similar models include fourth-order partial differential equation models (19) and combined models of second-order and fourth-order partial differential equation (20). However, although the pure high-order method effectively overcomes the step effect, it will cause the side effect of blurred edges. In order to balance these two aspects, a mixed-order method is proposed. In 2014, Niu et al. (21) proposed a penalty-weighted least squares method for sparse angle CT reconstruction based on the Generalized Total Variation (TGV) (22). In 2014, Hu et al. (23) proposed a generalized higher degree total variation (HDTV) regularization method. Many second-order TV extensions are special cases of generalized HDTV. The generalized HDTV significantly improves image quality, and increases the speed of the algorithm ten times.

In 2015, Cai et al. (24) proposed the TpV (Total p-Variation) model by replacing the l_1_-norm with the l_p_-norm for a better measurement of sparsity, which can solve the constrained optimization problems stably and effectively through the ADM method. In 2016, Zhang et al. (25) further proposed the TGpV regularization model, by using l_p_-norm to improve the sparsity of TGV. In 2019, Sun et al. (26) proposed a log-norm TV minimization, which uses non-convex log-norm instead of l_1_-norm to improve the TV minimization.

In the field of image denoising, the Low-Rank Matrix Approximation (LRMA) has attracted widespread attention. The LRMA can be solved by minimizing the nuclear norm, nuclear norm is the sum of the singular values of the matrix. In 2002, Nuclear Norm Minimization (NNM) (27) was proposed to solve LRMA, it was a convex relaxation of rank minimization. In 2012, Schatten p-norm minimization (28) was used to solve LRMA. However, both minimization techniques treat all singular values equally and shrink them with the same threshold, which will damage the integrity of edges and texture retention. In order to overcome this shortcoming, Gu et al. proposed the Weighted Nuclear Norm Minimization (WNNM) (29), and Xie et al. proposed the Weighted Schatten p-norm Minimization (WSNM) (30). Compared with the WNNM, the WSNM is a better approximation of the original LRMA problem. In (31), it was proved that when the weights are arranged in non-descending order, the WSNM can be equivalently transformed into a series of independent non-convex l_p_-norm sub-problems, and each sub-problem can be solved by the generalized soft-thresholding (GST) algorithm. In 2018, Zhang et al. (32) proposed the NOWNUM algorithm based on WNNM for sparse angle CT reconstruction.

In recent years, machine learning (33–35), especially deep learning (36–39), has aroused extensive research interest and made significant progress in many fields (40, 41), including medical imaging (42). In 2018, Chen et al. (43) proposed a new statistical iterative CBCT reconstruction algorithm based on neural networks, which uses a data-driven approach instead of manually designing penalty items to overcome the step effect and keep edge details. Zheng et al. (44) proposed a low-dose 3D CT reconstruction method based on learning and clustering. By using the penalty-weighted least square method, alternating between image reconstruction steps and clustering steps, their method optimizes cost functions and enhances image quality. In 2019, Jiang et al. (45) proposed a symmetric residual convolutional neural network SR-CNN based on deep learning, using a TV-based method to reconstruct CBCT from the limited projections simulated by real CT. Yang et al. (46) proposed a new neural network residual learning algorithm, which was directly applied to projection data to reduce the stripe artifacts in CBCT.

At the same time, many methods based on guided image filtering (GIF) were proposed for CT image reconstruction. In 2016, Ji et al. (47) proposed the SART-G algorithm, previous CT images are used as the initial prior guidance images for filtering guidance images. However, in most cases, previous CT images cannot be obtained. Therefore, how to carry out high-quality reconstruction without the help of previous CT images is of greater significance. In 2020, Shen et al. (48) proposed a guided image filter reconstruction based on TV and prior image (TVPI-G). This algorithm reduces the number of parameters and the influence of the prior image as early as possible in the iterative process. However, the best way to survey the GMI’s sparsity is to use the GMI’s l0- norm (49), TV is not the best choice (14).

GMI’s l_p_- norm has been shown as a better way to survey the GMI’s sparsity in TpV algorithm (24). Although the l_p_- norm causes non-convex optimization problems, the authors believe guided image filtering can help point out the iterative direction and find the right sparse solution. This paper proposes a sparse angle CBCT reconstruction algorithm based on guided image filtering (GIF), which can suppress noise and artifacts while well preserving edges and restoring texture. The GIF can transfer the features of the guidance image to the target image. Since SART has good edge retention ability and the TpV minimization can reduce noise, we use the TpV minimization result as the initial guidance image, and the SART result as the filter input. For each round of iteration, the guidance image is updated by the weighted average of the last guidance image and the SART reconstructed result. The experimental results based on simulated and real CBCT data prove the advantages of the combination of the two algorithms and the feasibility of guided image filtering in non-convex optimization problems.

## 2 Method

Guided Image Filtering (GIF) (50, 51) is a kind of edge-preservation smoothing operator, which has a better performance near the edges than the popular bilateral filters. Nowadays, the guided filter is both effective and efficient in a great variety of computer vision and computer graphics applications including noise reduction, detail smoothing, image defogging.

### 2.1 Guided Image Filtering Reconstruction Algorithm Based on Prior Image

The emergence of the guiding image filtering theory provides a new direction for sparse angle CT reconstruction. The SART-G algorithm (47) is one of them, which is based on SART and guiding image filtering. The algorithm sets the previously captured CT image as the initial prior guidance image for guided image filtering. Nowadays, the SART-G algorithm is mainly applicable to the situation where a prior image is known, and due to some small parts of the prior image having been changed, the sparse angle projection data is used to reconstruct the changed CT image.

The SART-G algorithm is an iterative algorithm. In the first iteration, the initial prior image is used as a guidance image to constrain the CT reconstruction. Later during each iteration, the guidance image is continuously updated. The updated guidance image combines the information contained in the initial prior guidance image and the image reconstructed by the SART algorithm, and the guided image filter conveys the combined information to the output, so that the changed part of the prior image can be effectively reconstructed. By considering the constantly updated guidance image, the effectiveness of the SART-G algorithm is ensured.

The CT scanning process can be discretized into the following linear system:


(1) y=Ax

Where ***A*** ∈ ***R*** *^M×N^*, ***A*** *_i,j_* = *a_i,j_* denotes the length of the ith X-ray through the jth image pixel. ***y*** is the projection data collected by the detector, ***x*** is the linear attenuation coefficient of the object.

In the SART-G algorithm. The iterative formula of SART is as follows:


(2) $$x_{j}^{k+1}=x_{j}^{k}+\lambda c_{j},c_{j}=\frac{\sum i\in I_{\varphi }\mu ia_{i,j}}{\sum i\in I_{\varphi }a_{i,j}},\mathrm{\mu i}=\frac{\hat{y}-y_{i}}{\sum_{j}^{N}=1^{a_{i,j}}}$$

The algorithm steps of the guiding image filtering are shown in **Table 1**.

**Table 1:** Guiding image filtering steps.

Input: input image P, guide image *I*, window radius R, regularization parameters ϵ
1 mean filter mean*_I_* = *f*_mean_ (*I*) ,mean*_P_ = f*_mean_ (*P*)
2 Calculate correlation coefficient cor *r* = *f*_mean_ (*I*· *I), corr_IP_ = *f*_mean_ (*I*· *P)
3 Calculate the variance var*_I_* = corr*_I_*_–_ mean*_I_* · *mean*_I_*
4 Computed covariance cov*_IP_* = cor *r_IP_* – mean*_I_* · *mean*_P_*
5 *a* = cov*_IP_* /(var + *ε*), *b* = mean*_P_* – *a*· *mean*_I_*;
6 mean*_a_* = *f*_mean_ (*a*), mean*_b_* = *f*_mean_ (*b*);
7 ***Q*** = mean*_a_* · *I + mean*_b_*
**output**: output image ***Q***

### 2.2 Sparse Angle CBCT Reconstruction Based on TpV Minimization

l_p_- norm is closer to l_0_- norm than l_1_- norm (24), which can better measure the sparsity of GMI. Using non-convex optimization and generalized p- shrinkage mapping, the TpV minimization model can be solved iteratively by alternating minimization (52).

The TpV minimization model is shown below:


(3) $$\arg\;\underset{z}{\text{min}}{\text{G}}_{p}(\text{z})\;s.t.\{\begin{array}{c} ||\text{y}-\text{Ax}|{|}_{2}^{2}=\text{e},||\text{e}|{|}_{2}^{2}\;\leq \epsilon \\ \text{z}=\nabla \text{x}\\ \text{x}\geq 0 \end{array}$$

*G_p_* (*z*) is a penalty function, whose proximal mapping ***S*** *_β,p_* is defined as:


(4) $$S_{\beta ,p}(x_{i})=s_{\beta ,p}(\left|x_{i}\right|)sign(x_{i})$$

Where S*_β_*,*_p_* (***x*** *_i_*) = max{*t* – *β^p–^*^2^*t^p–1^*,0}


$$z=\text{argmin }\|z{\|}_{p}^{p}+\frac{\beta }{2}\|z-\nabla f-r/\beta {\|}_{2}^{2},0<p\leq 1\;(5)$$

Where z is auxiliary variable, z = ^Δ^*&x*∈^3^*^N^*, and *r*∈^3N^ is an auxiliary variable of multipliers.

Then, ADM is used to solve the optimization problems with two separable variables. By using the augmented Lagrange function, Equation (3) can be expressed as the following unconstrained optimization problem:


(6) $$\begin{array}{l} L_{A}(\text{x},\text{z})=G_{p}(z)-\lambda_{j}^{T}(\text{z}-\nabla \text{x})+\frac{\beta_{1}}{2}|\text{z}-\nabla \text{x}{|}_{2}^{2}\\ -\lambda_{j}^{T}(y-Ax-e)+\frac{\beta_{2}}{2}|y-\text{Ax}-\text{e}{|}_{2}^{2}+\delta_{\text{pos}}(\text{x})\\ \delta_{\text{pos}}(\text{x})=\{\begin{array}{l} 0,\text{x}\in {\mathbb{R}}_{+}^{\text{N}}\\ \infty ,\text{x}\notin {\mathbb{R}}_{+}^{\text{N}} \end{array} \end{array}$$

The question is split into two subproblems of x and z, and then iterated alternately.

The algorithm steps of the TpV minimization model are shown in **Table 2**.

**Table 2:** TpV minimization algorithm steps.

Input: projection matrix A, projection data vectory, β_1_, β_2_, η, ϵ, initial value z^(0)^, x^(0)^ let *k* = 0
1 Iterate the following steps until the iteration termination condition is met
2 Update $z^{\left(k+1\right)},\;z^{\left(k+1\right)}\leftarrow S_{p}(\nabla x^{\left(k\right)}+\lambda_{1}^{\left(k\right)}/\beta_{1})$
3 Update $x^{\left(k+1\right)},\;{\text{x}}^{\left(k+1\right)}\leftarrow \text{pos}\left\{\text{F}\left[{\text{F}}^{-1}\left[\nabla^{\text{T}}\left[{\text{β}}_{1}{\text{z}}^{\left(\text{k}+1\right)}-{\text{λ}}_{1}^{\left(\text{k}\right)}\right]+{\text{A}}^{T}{\text{λ}}_{2}^{\left(\text{k}\right)}+\frac{{\text{β}}_{2}}{\text{τ}}{\text{x}}^{\left(\text{k}\right)}-{\text{β}}_{2}{\text{d}}^{\left(\text{k}\right)}\right]/\text{J}\right]\right\}$
Where function *pos* forces all elements with negative values to be 0.
4 Update $e^{\left(k+1\right)},\;e^{\left(k+1\right)}\leftarrow \min\;\left\{1,\epsilon /{\left\|y-Ax^{\left(k+1\right)}\right\|}_{2}^{2}\right\}\cdot (y-Ax^{\left(k+1\right)}ht)$
5 Update $\lambda_{1}^{\left(k+1\right)},\;\lambda_{1}^{\left(k+1\right)}\leftarrow \lambda_{1}^{\left(k\right)}-\eta \beta_{1}\left(z^{\left(k+1\right)}-\nabla x^{\left(k+1\right)}\right)$
6 Update $\lambda_{2}^{\left(k+1\right)},\;\lambda_{2}^{\left(k+1\right)}\leftarrow \lambda_{2}^{\left(k\right)}-\eta \beta_{2}\left(y-Ax^{\left(k+1\right)}-e^{\left(k+1\right)}\right)$
7 *k* & *k* + 1
8 End
**output:** reconstruction result *x^(k)^*

### 2.3 Guided Image Filtering Reconstruction Based on TpV Minimization

Inspired by the SART-G algorithm, this section proposes the TpV-GIF algorithm, which uses the TpV minimized reconstruction result as the initial guidance image, and dynamically updates the guidance image during iterations. In the initial iteration, the guidance image mainly reflects TpV’s output to quickly remove the noise in the SART’s result. As the iteration progresses, the noise in the SART reconstruction result is effectively removed, at this time, the proportion of TpV reconstruction result in the guidance image is reduced to avoid over-smoothing, turning to focus on preserving edges and texture. The steps of the TpV-GIF algorithm are as follows:

1. Parameter initialization, projection matrix A, projection data y, the number of iterations N, the initial value of the image *f*^(0)^, and the parameters of the guide image filtering step: window radius R, regular term parameters ϵ;

2. Calculate the initial guided image *I^initial^*, which is the reconstruction based on the minimization of TpV (see **Table 2**);

3. For the nth iteration, using *f*^(n-1)^ as the initial value of the SART algorithm iteration to perform SART algorithm reconstruction, the reconstruction result is $f_{SART}^{\left(n\right)}$ ;

4. Update the guide image *I^guide^*:


(7) $$I^{guide}=I^{initial}\times (N-n)/N+f_{SART}^{\left(n\right)}\times (n/N)$$

5. Take $f_{SART}^{\left(n\right)}$ as the filtered input image, and use *I^guide^* as the guiding image for guiding image filtering; the output image is $f_{SART-G}^{\left(n\right)}$ (see **Table 1**);

6. $f^{\left(n\right)}=f_{SART-G}^{\left(n\right)},\;n=n+1$ ;

7. Repeat steps (3)-(6) until the iteration termination condition is met, and the reconstruction result is obtained.

In this paper, for the TpV minimization algorithm, p is set to 0.9. For the guidance image filtering step, the guided image filter operates the CBCT image layer by layer, the window radius is set to 4, and the regularization parameter ϵ is set to 0.0016.

## 3 Dataset

We first use the digital brain model (53) for reconstruction as the data 1. The model is created based on realistic MRI data of the human brain. It is widely used model for cone beam CT reconstruction and very suitable for evaluating reconstruction.

Then, we use real clinical projection data for reconstruction as data 2. In the dataset, the distance from the source to the detector is 1040mm, and the distance from the source to the origin is 570mm. We use projection data from 32 angles for reconstruction. The size of reconstructed voxels is 256×256×266, and the voxel resolution is 1.6272mm×1.6272mm×1mm.

## 4 Experiment and Results

### 4.1 CBCT Reconstruction Quality Evaluation Index

This paper uses Root Mean Square Error (RMSE), Peak Signal to Noise Ratio (PSNR) and Structural Similarity Index (SSIM) as evaluation indicators, to quantitatively nalyse and compare the reconstruction results.

The RMSE is the square root of the mean square error (MSE). MSE is the ratio of the sum of squares of the voxel value errors between the reconstructed image and the true value image to the number of voxels (54, 55), defined as the formula (2):


(8) $$\text{MSE(}\hat{x},x)=\frac{1}{N}\sum_{i=1}^{N}{\left({\hat{x}}_{i}-x_{i}\right)}^{2}$$

Among them, $\hat{x}$ is the reconstructed image, x is the actual image, $\hat{x}$ and *x_i_* are the element values of the reconstructed image and the actual image, and N is the number of image elements. The definition of RMSE is shown in formula (3):


(9) $$\text{RMSE(}\hat{x},x)=\sqrt{\text{MSE(}\hat{x},x)}$$

RMSE is used to measure the deviation between two signals (56). Generally, the smaller the value of RMSE, the better the reconstruction result. PSNR is also a common objective standard for evaluating images (57), and its definition is shown in formula (4):


(10) $$\text{PSNR(}\hat{x},x)=10{\text{log}}_{10}\left(\frac{2^{l}-1}{MSE}\right)$$

Where *l* is the bit depth of the image. For example, when the pixel value range is 0-1, the value of *l* is 1, and when the pixel value range is 0-255, the value of *l* is 8. Both RMSE and PSNR are based on the deviation between pixel values and do not consider the visual characteristics of the human eye, so the results may be inconsistent with human subjective perception.

SSIM measures the structural similarity between two images (58), and its definition is shown in formula (5):


(11) $$\text{SSIM(}\hat{x},x)=\frac{\left(2\mu_{\hat{x}}\mu_{x}+c_{1})(2\sigma_{\hat{x}x}+c_{2}\right)}{\left(\mu_{\hat{x}}^{2}+\mu_{x}^{2}+c_{1})(\sigma_{\hat{x}}^{2}+\sigma_{x}^{2}+c_{2}\right)}$$

Among them, $\mu_{\hat{x}}$ and *μ_x_* are the pixel mean values of the reconstructed image and the real image respectively, $\sigma_{\hat{x}}$ and *σ_x_* are the variances of the reconstructed image and the real image *resspectively*, and $\sigma_{\hat{x}x}$ is the covariance. And *c*_1_ are *c*_2_ two very small constants, mainly to prevent the denominator from being zero.

In addition, the Profile curve method is also a common method for judging the quality of the reconstructed image. It selects a certain row or column of the image, takes the pixel position as the abscissa and the pixel value as the ordinate, and draws the gray-scale curve of the reference image and the reconstructed image in a picture. By observing the similarity between the curve and the reference image curve, the quality of the reconstruction result can be easily judged.

### 4.2 Simulation Model Reconstruction

We use the Siddon line driver to simulate the projection data, generate projection data from 32 angles, and use these projection data for reconstruction. This paper selects 4 widely used methods (SART, ASD-POCS, SART-TV, TpV) as the comparison algorithm, where SART and TpV can be found in section 3, ASD-POCS is an algorithm who combines the ART algorithm and TV algorithm, SART-TV combines the SART algorithm and TV algorithm.

**Figure 1:**
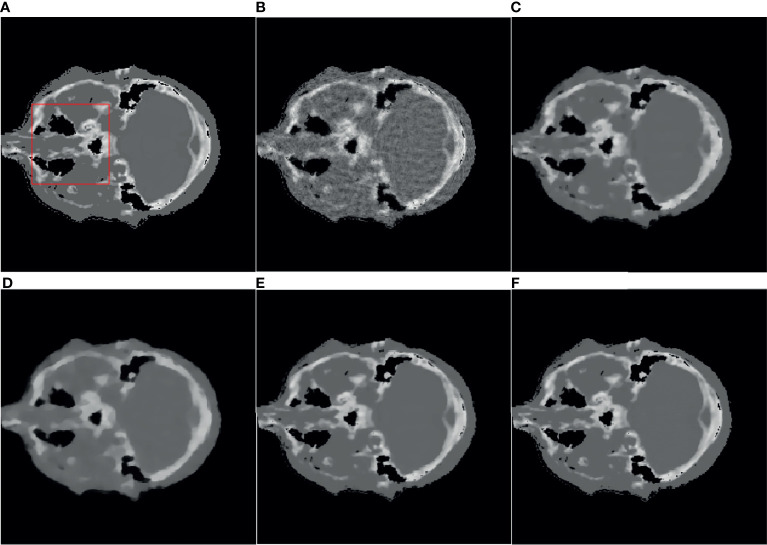
Reference image and 32-angle reconstruction results: **(A)** reference image; **(B)** SART; **(C)** ASD-POCS; **(D)** SART-TV; **(E)** TpV; **(F)** TpV-GIF.

For the SART algorithm, λ is set to 1, the reduction of λ, *λ_red_* is set to 0.99. For the ASD-POCS algorithm, λ is set to 1, *λ_red_* is set to 0.99, TV hyperparameter α is set to 0.002. For the SART-TV algorithm, λ is set to 1, λ_TV_ is set to 15 which gives the ratio of importance of the image vs the minimum total variation. and the reconstruction result of the 80th layer of data 1 is shown in **Figure 1**.

**Figure 2:**
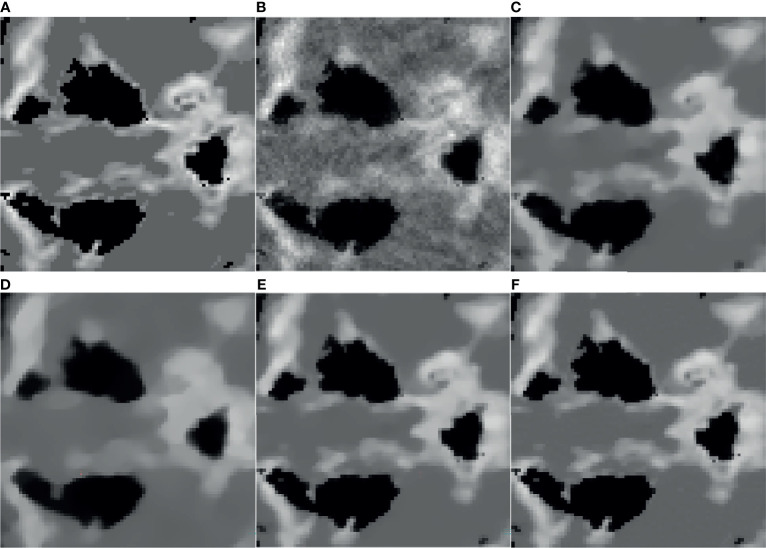
ROI area enlargement result: **(A)** real graphics; **(B)** SART; **(C)** ASD-POCS; **(D)** SART-TV; **(E)** TpV; **(F)** TpV-GIF.

**Figure 3:**
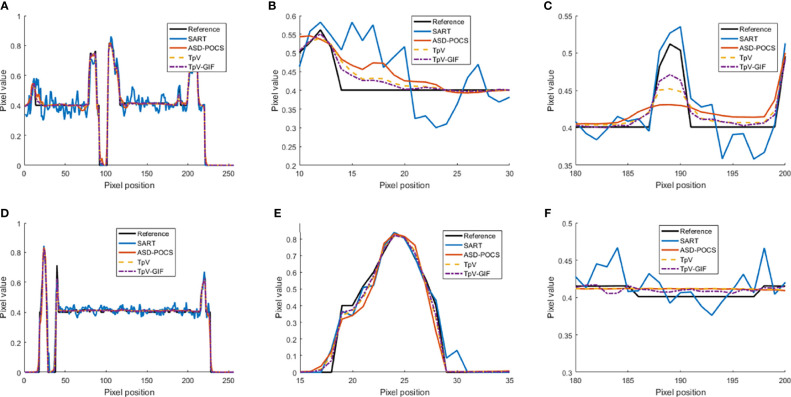
Profile curve images: **(A)** The full voxel curve of the 80th layer and 128 rows; **(B, C)** is a partial enlargement of **(A)**; **(D)** the complete curve of the 128th row and 128 columns; **(E, F)** Is a partial enlargement of **(D)**.

It can be seen from **Figure 1B** that the SART reconstructed image under the sparse angle is full of artifacts and the image quality is poor. The images in **Figures 1C-F** are relatively smooth because they all involve TV constraints. However, the TV minimization reconstruction result in **Figure 1C** has a step effect, and many small details are smoothed out. The image in **Figure 1D** is more serious in the problem of excessive smoothness and has a strong sense of smearing. The image reconstructed based on TpV minimization is much better, suppresses artifacts while retaining most of the edges and details, and the reconstruction results of the algorithm proposed in this paper show better edge retention characteristics. In order to better observe the reconstruction details, the part of the image in the red box in **Figure 1** is individually enlarged and displayed to **Figure 2**. As shown in **Figure 2**, our algorithm can effectively remove artifacts while better restoring the edges and details of the image. **Figure 3** is a profile graph. It can be seen that the reconstruction result of TpV-GIF is very close to the reference image, no matter in a smoother area or an edge area where the voxel value changes greatly. This means that the algorithm has a good performance in suppressing noise and preserving edge details.

**Table 3** shows the quantitative evaluation results of CBCT reconstruction of the entire model. It can be seen from **Table 4** that, compared with other algorithms, the TpV-GIF we proposed has obtained the highest score in the quantitative evaluation index.

**Table 3:** Quantitative evaluation of brain phantom reconstruction with 32-angle projection.

	RMSE	PSNR	SSIM
SART	0.0219	33.1911	0.9855
ASD-POCS	0.0227	32.8975	0.9846
SART-TV	0.0312	30.1169	0.9781
TpV	0.0158	36.0269	0.9883
TpV-GIF	**0.0114**	**38.8500**	**0.9900**

Bold values are the results of our method, RMSE, PSNR,SSIM have been shown in equation 9-11.

**Table 4:** Quantitative evaluation of 32-angle projection real projection data reconstruction.

	RMSE	PSNR	SSIM
SART	0.0354	29.0199	0.9743
ASD-POCS	0.0404	27.8724	0.9684
SART-TV	0.0443	27.0719	0.9653
TpV	0.0258	31.7568	0.9777
TpV-GIF	**0.0184**	**34.7036**	**0.9814**

Bold values are the results of our method, RMSE, PSNR,SSIM have been shown in equation 9-11.

### 4.3 Actual Data Reconstruction

**Figure 4:**
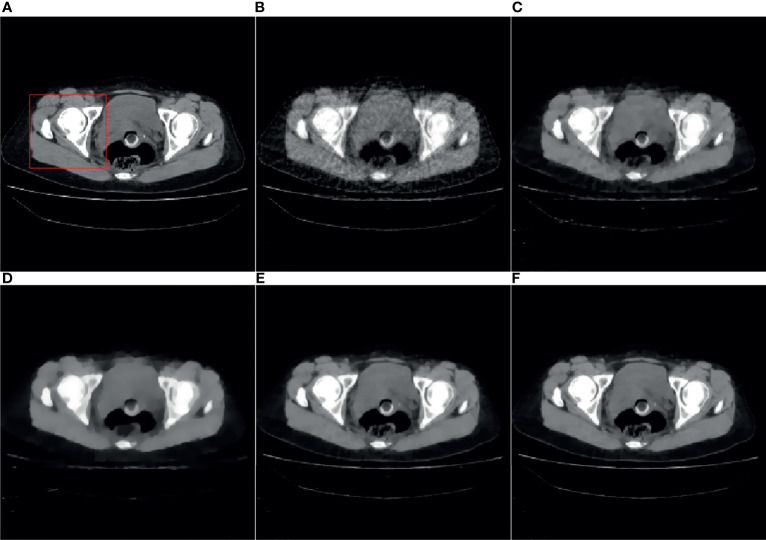
40th slice reconstruction result: **(A)** reference image; **(B)** SART; **(C)** ASD-POCS; **(D)** SART-TV; **(E)** TpV; **(F)** TpV-GIF.

**Figure 5:**
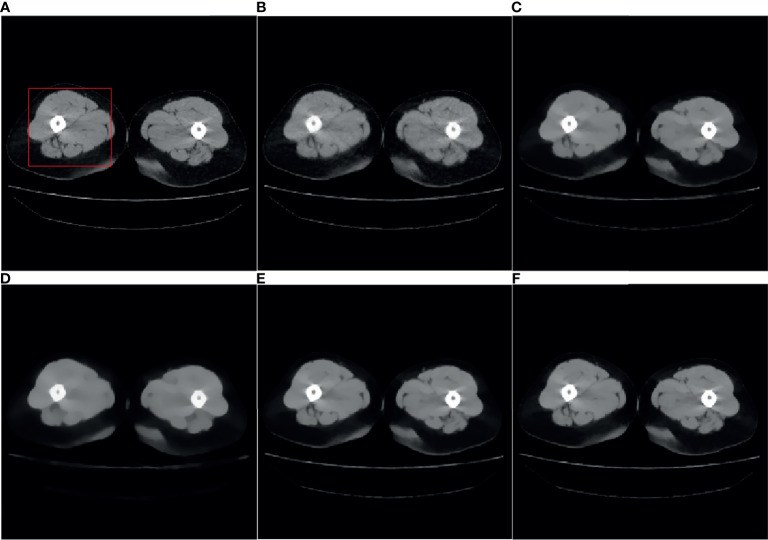
260th slice reconstruction result: **(A)** reference image; **(B)** SART; **(C)** ASD-POCS; **(D)** SART-TV; **(E)** TpV; **(F)** TpV-GIF.

**Figure 6:**
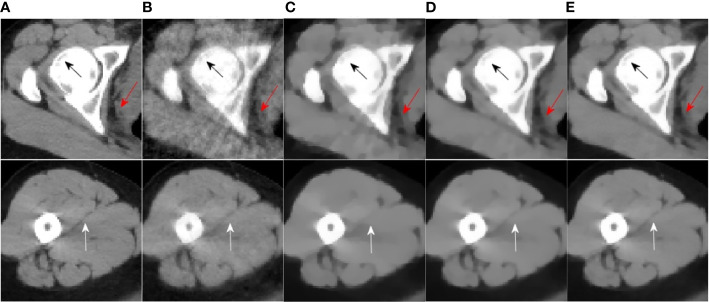
ROI area enlarged image: the first row and the second row are the enlarged images of the red box area in **Figure 4** and **Figure 5**, from left to right there are the reference image, SART, ASD-POCS, TpV and TpV- Reconstructed image of GIF. The **(A–E)** in this figure are correspond to the **(A–E)** in **Figure 4** and **Figure 5**.

**Figures 4**, **5** are the reconstruction results of CBCT of the place that close to the center plane and bottom plane, respectively, it can be seen from **Figure 4B** that with the sparse angle projection, the reconstruction result of SART near the center plane still has serious artifacts. The reconstruction result of the SART-TV algorithm has a significant blocky effect, and many details are blurred. The reconstruction result of ASD-POCS algorithm is better than that of SART-TV reconstruction, but compared with the TpV minimization algorithm, the blocking effect is still more serious. The reconstruction result of the TpV-GIF algorithm not only suppresses artifacts as effectively as TpV, but also has better detail restoration, which can be seen in **Figure 6**.

Observe the details indicated by the black and white arrows in **Figure 6**, and compared with the reference image, you can see that the ASD-POCS reconstruction result is relatively fuzzy, and details of the SART reconstruction image are more complete, but they all have serious artifacts. The details of the TpV reconstruction image are better preserved while the performance of TpV-GIF is even better. For the area indicated by the red arrow, the SART does not perform well in suppressing artifacts, the ASD-POCS blurs the details and has a blocky effect, on the contrary, TpV and TpV-GIF reconstruction results are better, the artifacts are suppressed while the edges are preserved.

**Figure 7:**
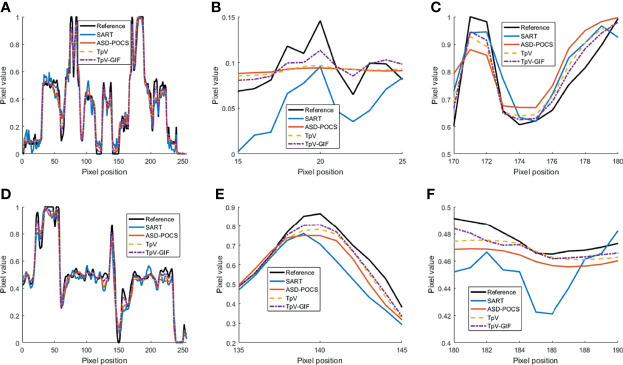
Profile curve images. **(A)** The full voxel curve of the 128th row of the 133rd slice; **(B, C)** A partial enlargement of **(A, D)** the axial voxel curve of the 128th row and 128th column of all faults; **(E, F)** Partial enlargement of **(D)**.

**Figure 8:**
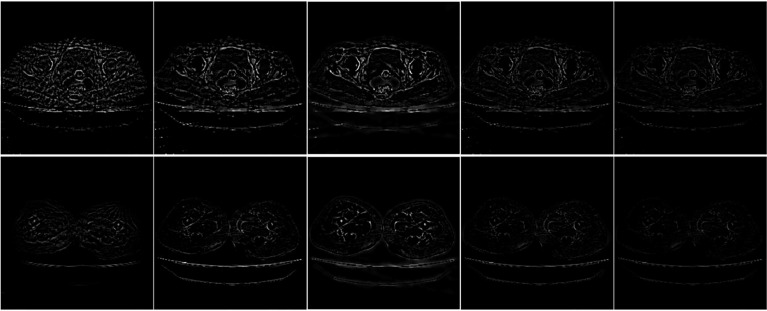
The absolute difference between the reconstructed image and the reference image.

From **Figure 7**, it can be seen that the SART reconstruction results are very smooth regardless of whether it is in a smooth area or an area where the voxel value changes greatly, so the edges and details may be blurred. The SART reconstruction result also changes seriously in the smooth area, which is reflected in the image as an artifact. TpV and TpV-GIF are relatively smooth in the smooth area, and change with the true value in the area where the voxel value changes greatly. The TpV-GIF curve is closest to the reference image curve, which means that the reconstruction result of the TpV-GIF algorithm is more accurate. **Figure 8** shows the absolute difference between various reconstruction algorithms and the reference image. Obviously, the difference between the TpV-GIF reconstructed image and the reference image is smaller.

**Table 4** shows the quantitative evaluation results of the overall reconstruction of the real clinical data. It can be seen from **Table 4** that the TpV-GIF algorithm has achieved the best results among the three evaluation criteria, which is consistent with the results of the visual evaluation.

## 5 Conclusion

Aiming at solving the problem of incomplete projection data under sparse angle projection, this paper proposes and discusses a better reconstruction algorithm based on guided image filtering. Guided image filtering is a kind of edge-preserving filtering that utilizes the guidance image to effect the result of the output image, transfers the characteristics of the guidance image to the output image. We combine the SART with edge preservation characteristics and the TpV minimization with good smoothing characteristics and capable of suppressing artifacts. The SART reconstruction result is used as the filter input, and the reconstruction results of the two algorithms are combined as the guidance image and are dynamically updated. Verifications and comparisons performed using various datasets illustrate that the proposed method is effective and promising.

## Data Availability Statement

Publicly available datasets were analyzed in this study. This data can be found here: The data used in this paper is an open-source data provided by the Hamlyn Center at Imperial College London at hamlyn.doc.ic.ac.uk/vision (accessed on 9 November 2021).

## Author Contributions

Conceptualization, WZ and BY; methodology, BY and SX; software, SX; validation, CX and CL; formal analysis, JT; investigation, YL and LY; resources, JT and LY; data curation, SL and SX; writing—original draft preparation, YL and JT; writing—review and editing, SL; visualization, YL and LY; supervision, BY; project administration, WZ; funding acquisition, WZ. All authors have read and agreed to the published version of the manuscript.

## Funding

This research was funded by the Sichuan Science and Technology Program, grant number 2021YFQ0003.

## Conflict of Interest

The authors declare that the research was conducted in the absence of any commercial or financial relationships that could be construed as a potential conflict of interest.

## Publisher’s Note

All claims expressed in this article are solely those of the authors and do not necessarily represent those of their affiliated organizations, or those of the publisher, the editors and the reviewers. Any product that may be evaluated in this article, or claim that may be made by its manufacturer, is not guaranteed or endorsed by the publisher.
